# *Scedosporium apiospermum* brain abscesses in a patient receiving ibrutinib and venetoclax

**DOI:** 10.1002/jha2.121

**Published:** 2020-10-26

**Authors:** Rocio Figueroa, Nicolas Martinez‐Calle, Katie Prescott, Mark Bishton

**Affiliations:** ^1^ Clinical Haematology Nottingham University Hospitals NHS Trust; ^2^ Pathology Nottingham University Hospitals NHS Trust

**FIGURE 1:**
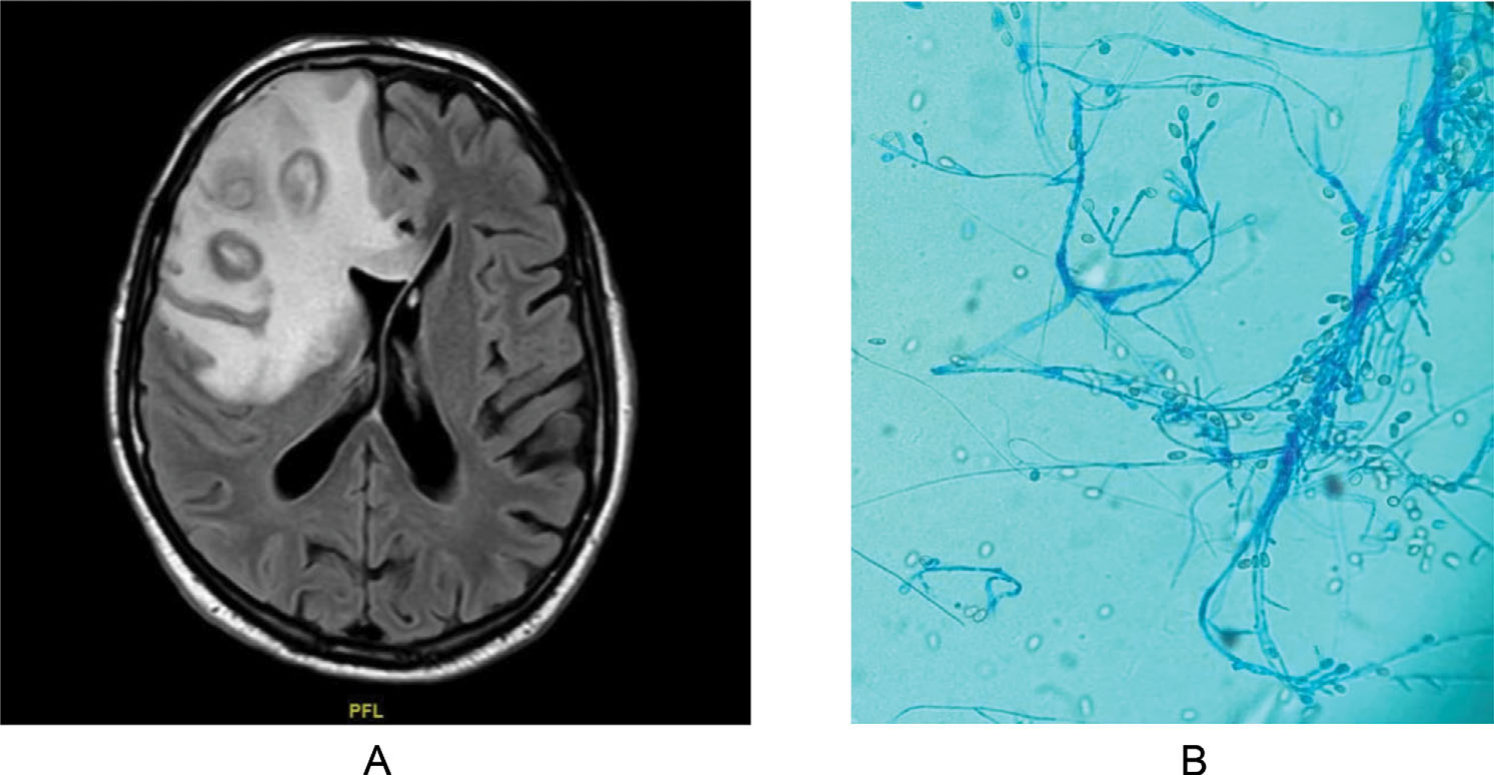
A. Long TR‐FLAIR sequence Head MRI showing multiple right frontal lobe abscesses. B. Microscopic picture of *Scediosporium apisopermum* obtained from surgical drainage.

An 80‐year‐old male, with relapsed mantle cell lymphoma was treated on a trial with the combination of the bruton tyrosine kinase (BTK) inhibitor, ibrutinib, and BH3‐mimetic, venetoclax, and achieved complete remission by both Computerized Tomography‐Positron Emission Tomography (CT‐PET) and molecular assessment. Despite a persistent mild neutropenia noted at study visits, which did not require drug interruption or Granulocyte‐colony stimulating factor (GCSF) by protocol, he had a normal IgG level and had not suffered any infectious complications during 2 years of therapy. Following a fall at home, he presented to hospital with confusion. CT and Magnetic Resonance Imaging (MRI) head demonstrated multifocal right frontal lobe brain abscesses requiring urgent neurosurgical drainage (Figure 1A). *Scedosporium apiospermum* was cultured from pus (Figure 1B). The isolate was sent to the Public Health England mycology reference laboratory where in vitro sensitivity to amphotericin B and voriconazole was demonstrated. The patient had no other attributable risk factors such as near drowning or prolonged steroid therapy. CT‐PET did not demonstrate any evidence of systemic fungal infection and confirmed ongoing metabolic remission. Both ibrutinib and venetoclax therapy were discontinued and the patient was commenced on therapeutic voriconazole, with close therapeutic drug monitoring. Clinical and radiological improvement was observed, and the patient remains clinically well 4 months later.

*Scedosporium apiospermum* is a filamentous fungus found in soil and stagnant water. It can result in severe disseminated infections in immunocompromised hosts, most commonly following accidental inhalation of contaminated water. Common sites of infection include lung, bone, and the central nervous system. Treatment of these infections is challenging in view of resistance to many antifungal agents.

In addition to the inhibition of BTK in multiple immune effector cells, ibrutinib also irreversibly inhibits BTK in T cells, with resulting inhibition of T‐ and NK‐cell function and impaired macrophage phagocytosis. Opportunistic infections such as *Aspergillus* and *Pneumocystis* have been reported with ibrutinib both as monotherapy and in combination, and herpes simplex and *Pneumocystis jirovecii* prophylaxis are considered mandatory. In contrast, opportunistic infections secondary to venetoclax monotherapy appear rare. To our knowledge, central nervous system infection due to *S. apiospermum* has not been previously reported. Given limited published data regarding the risk of serious infection with ibrutinib/venetoclax, our case is a cautionary tale that severe infections associated with molecular targeted "chemo free" agents may occur in the absence of other risk factors and after prolonged therapy.

